# The digitalisation of finance management skills in dementia since the COVID-19 pandemic: A qualitative study

**DOI:** 10.1177/14713012231159156

**Published:** 2023-02-20

**Authors:** Clarissa Giebel, Kath Halpin, Jean Tottie, Lena O’Connell, Joan Carton

**Affiliations:** Department of Primary Care and Mental Health, 4591 University of Liverpool, Liverpool, UK; NIHR Applied Research Collaboration North West Coast, Liverpool, UK; NIHR Applied Research Collaboration North West Coast, Liverpool, UK; TIDE (Together In Dementia Everyday), Liverpool, UK; Department of Primary Care and Mental Health, 4591 University of Liverpool, Liverpool, UK; NIHR Applied Research Collaboration North West Coast, Liverpool, UK; NIHR Applied Research Collaboration North West Coast, Liverpool, UK

**Keywords:** COVID-19, dementia, everyday activities, finance management

## Abstract

**Objectives:**

Engaging with finances has become increasingly digitalised in recent years, particularly since the COVID-19 pandemic, yet it is unknown how finance management has been affected in people living with dementia. The aim of this qualitative study was therefore to explore how digitalisation and the recent pandemic have affected finance management skills in people with dementia.

**Methods:**

Semi-structured interviews were conducted remotely with people with dementia and unpaid carers living in the UK via phone or Zoom between February and May 2022. Transcripts were coded by one of four different research team members, including two unpaid carers who were public advisers on the project. Data were analysed using inductive thematic analysis.

**Results:**

Thirty carers and people with dementia participated, and five overarching themes were identified. Managing finances has been both simplified and made more complicated by digitalising how money is spent and managed, with people with dementia and unpaid carers reporting advantages of using direct debits and debit cards, as well as digital illiteracy barriers for older relatives with dementia. Unpaid carers have received no support in managing their relative’s finances, and were found to be burdened by the additional caring duties.

**Conclusions:**

Carers need to be supported in managing their relative’s finances as well as with their general well-being due to taking on additional caring duties. Digital systems for finance management need to be user-friendly for people with cognitive impairment, with a need for digital literacy training for middle-aged and older adults to avoid difficulties if they develop dementia, and improved access to a computer/tablet/smart phone.

## Introduction

Across the globe, over 55 million people are estimated to live with dementia (World Health Organisation [WHO], 2021), with unpaid carers representing the backbone of care. Unpaid carers are often not recognised for their contributions, although they provide care worth £13.9 billion each year alone in the UK for example (Wittenberg, Hu, Barraza-Araiza, & Rehill, 2019).

The ability to perform more complex instrumental activities of daily living (IADLs), such as engaging in hobbies, preparing a meal, managing medication, or preparing a hot drink, deteriorates more quickly than the ability to perform more basic forms of activities of daily living (ADLs), such as bathing, dressing, and feeding (Roehr et al., 2019). Specifically, finance management skills are reported to be the first instrumental activity of daily living (IADL) to deteriorate in dementia (Peres et al., 2008). Peres et al. (2008) showed how finance management skills have been found to deteriorate up to 10 years prior to a diagnosis. Subsequent research has further shown how the initiative, not only the performance, to engage in finance management tasks deteriorates early on in dementia (Giebel, Sutcliffe, & Challis, 2017), with wider finance management abilities linked to executive functioning (Giebel, Flanagan, & Sutcliffe, 2019). Deteriorations in finance management ability are not only common in dementia, but have also been reported in other neurodegenerative conditions including mild cognitive impairment and Parkinson’s disease (Bangma, Tucha, Tucha, De Dey, & Koerts, 2021), and difficulties can cause increased levels of stress in unpaid carers (Wawrziczny, Duprez, & Antoine, 2020).

Finance management, or financial capacity as coined by some, can be broken down into individual tasks. For quantitative assessments, the Financial Capacity Instrument (FCI) (Marson, Sawrie, & Snyder, 2000) provides a breakdown of six individual tasks and subtasks, including basic monetary skills, financial conceptual knowledge, cash transactions, cheque book management, bank statement management, and financial judgement. In a systematic review reporting on 10 studies, Sudo and Laks (2017) showed how basic skills remained more preserved in the early stages of dementia, whilst more complex finance skills had deteriorated early on. However, the six finance skills laid out in the FCI are outdated to modern standards, especially with the digitalisation of finances, which has been further advanced since the pandemic. Thus, there is no evidence on modern approaches to finance management in dementia, to date.

Increased digitalisation before and since the pandemic has not only affected how finances are dealt with in general, but the necessity of handling everything remotely since COVID-19 has severely impacted on dementia care more broadly. Vital social care and social support services, including day care centres, respite care, paid home care, and support groups, nearly all closed down at the beginning of the pandemic and were very slow in reopening or providing digital support, where feasible (Giebel et al., 2021). However, the provision of digital remote support only precludes some people with dementia and unpaid carers who have no access to a computer or tablet, or internet, causing exacerbated inequalities in accessing dementia care. This had a severe negative impact on the care people with dementia, and unpaid carers, could receive, and engaging in digital support did not offer the same benefits (Giebel et al., 2022). Whilst evidence has highlighted the wider impact of the pandemic on living with dementia, no focus has been placed on a key autonomous daily activity in dementia which not only focuses on how money is dealt with, but also on paying for care and managing finances in the long-term.

The aim of this qualitative study was therefore to explore how digitalisation and the recent COVID-19 pandemic have affected finance management skills in people with dementia, and its associated impact on unpaid carers. Whilst a growing body of evidence reports on the impact of the pandemic on people with dementia more broadly, including reduced access to care and poorer mental well-being (Lion et al., 2022; West et al., 2021), the increased digitalisation of care and everyday lives is likely to have influenced finance management skills and spending habits in people living with dementia. However, there appears to be no evidence to date on these skills and parts of daily life/instrumental activity of daily living.

## Methods

### Participants and Recruitment

Ethical approval was obtained from the University of Liverpool Ethics committee prior to the study beginning [Ref: 10612]. Participants (unpaid carers and people with dementia aged 18+) were recruited via Third Sector organisations, social media, the NIHR Applied Research Collaboration North West Coast, and the Liverpool Dementia & Ageing Research Forum. In addition, the study was registered with the Join Dementia Research online service which matched research volunteers with the study.

People with dementia had to have mental capacity to be eligible to participate. This was assessed by the postdoctoral researcher (LOC) who has had training in capacity assessment, which was based on the Mental Capacity Act (2005), and has assessed capacity in previous research.

### Data Collection

Semi-structured interviews were conducted remotely via Zoom or telephone. Prior to the interview, verbal informed consent was obtained and recorded, and for people with dementia, their mental capacity to participate was assessed.

Interview topic guides were co-developed with two unpaid carers who were equal team members (JC, KH). The topic guide (see Appendix) included questions about the ability to manage finances, how money was spent, how the pandemic had affected spending habits, as well as questions about paying for care services and legal matters, including Power of Attorney.

Interviews were audio recorded via Zoom and transcribed using Otter.ai software. A research team member who conducted the interviews (LOC) checked through the transcripts and filled in any missing and not transcribed words whilst fully anonymising each transcript.

### Data Analysis

Anonymised transcripts were coded by one of the research team members (CG, LOC, KH, JC), including the public advisers. Public advisers received training to conduct the analysis on two transcripts each. Data were analysed using inductive thematic analysis (Braun & Clarke, 2006) using the six-step approach. At the start of the process, the members of the research team familiarised themselves with the data by reading and re-reading the transcripts and writing down the initial codes. Following that, the initial codes were discussed and clarified at team meetings and emerging themes were identified, with all research team members, including the public advisers, agreeing on the identified themes for this analysis. The themes were later reviewed, defined and named, with four selected for inclusion in the manuscript.

### Public Involvement

Two unpaid carers for people living with dementia (JC, KH) were involved in all aspects of this study. Both attended regular team meetings, provided their own insights from their caring experiences to shape the research and interpret the findings, jointly developed the topic guides with the team, coded two transcripts each after training, and embedded the findings into their experiences of caregiving. Both read through drafts of this manuscript and will develop a lay summary based on these findings. In addition, a third unpaid carer who was part of the steering committee of the wider study (JT) helped interpret the findings and read through drafts of the manuscript. All activities were reimbursed according to NIHR INVOLVE guidance.

## Results

Thirty unpaid carers and people living with dementia participated in the study. Of these, 23 were carers and seven were people living with dementia. Carers were on average 64 (±8) years old [Range: 48–81], and the majority were female (74%; *N* = 17). One interview was conducted with a husband and wife who were former carers for the husband’s mother. Eight were daughters and one was a son caring for a parent, 10 were spouses, one carer looked after her sister, and two female carers looked after their partners, one current and one former.

Of the seven participants living with dementia, three were female and four were male, with an average age of 70 (±12) years [Range: 52–88]. Four lived alone, two lived with their spouses and one with her son.

### Qualitative Findings

Analysing the data using inductive thematic analysis resulted in five overarching themes, with different subthemes: The potential dangers of losing finance management skills early on; Face-to-face shopping skills and experiences; Moving to digital: Barriers and facilitators; Fast-tracked digitalisation due to COVID-19; Impact on carers supporting someone with finances (Table 1).

**Table 1.** Quotes by themes and sub-themes.

Theme/Subtheme	Quotes
Theme 1: The potential dangers of losing finance management skills early on	
	*“he was very meticulous in keeping his accounts and everything else. And suddenly, well, gradually, it became complete disarray. And you’d have papers everywhere, not understanding what was there. And this is his money. It was one of the early warnings that I didn’t actually recognise at the time, I thought I didn’t clue it into dementia or anything.” (ID18, female carer, 76)* *“And that’s one of his big things that he likes to do that he enjoys doing. […] Not that there’s any great amount of interest, but, you know, he knows that his pension’s going in. […] But the worst thing probably is the fact that most of its online. And he forgets his passwords, and gets locked out a lot. So I seem to be forever either resetting them, [or say] don’t let it do it three times wrong. You know, just wait for me to be home or what have you, those are some of the issues. I mean compared to a lot of people of his age, he still manages remarkably well with online banking. But a lot of it now is jointly managed or controlled by myself, to help him really in keeping calm about it.” (ID22, female carer, 64)*
Theme 2: Face-to-face shopping skills and experiences	
Lack of support and recognition from other shoppers and staff	*“It’s [shopping in-person] very difficult with dementia, because it was [husband’s] behaviour that changed. And it’s not the actual banks or shops themselves, it’s the customers within those areas who are, well, some, frankly, were very rude and aggressive and impatient with him. One pushed a trolley towards him, nearly knocked him over… somebody was excessively rude and then [husband] was in tears in the middle of the store”. (ID18, Female Carer, 76)* *“But the customers need dementia awareness, as I mentioned. You know, it was very important to me that I was able to take him out to do things as long as I could keep him into a routine.” (ID18, Female, Carer, 76)* *“I think the biggest issue I had… was when we first started doing the shopping during the pandemic… We got to the till, we were sort of like keeping our two metre distance. So the cashier was serving the woman in front of us and we were stood out two metres back. And then she shouted out, “Are you two together?” And I said, “Yes.” And then she said, “Well, you shouldn’t be in here. Can one of you go and stand over there?” So I had to sort of like shout in front of everybody, “My husband has dementia!” And so pretty much that’s been the worst. You know, the worst case I’ve had to deal with.” (ID21, Female, Carer, 62)*
Feeling rushed and anxious	*“There are some other reasons to pay by card because it’s easier. I don’t have to worry about holding up the queues, and people are impatient, and I notice it more since I’ve been disabled that people just are not kind, especially with the pandemic because it hasn’t really helped either… If I have to go shopping in the supermarket, I go to self-service so that I don’t hold up anybody. I can be, I can take my time and there’s nobody tutting or being behind me, or something.” (ID2, Female, PLWD, 52)* *“It’s the people being impatient… So however much, you know, when you pressured at the checkout, I will not leave the checkout, I’ve got to say. I don’t care what anybody thinks of me”. (ID26, Female, PLWD, 62)* *“So unfortunately, [the experience of being accused of shoplifting] left me a little bit fearful. I know it sounds stupid. It’s in the back of my mind that I might do it again. Yes, I’ve no financial problems, I have a debit card, credit card, no financial problems. Never shoplifted in my life before, it’s to do with the illness not me. It just completely went out of my mind to pay… And so it makes me a little bit nervous.” (ID10, Male, PLWD, 72)* *“The consultant kindly gave me a letter to carry around with me that this [forgetting to pay for the shopping] can possibly happen through the dementia. So if it ever happens again, I’ve got some proof, together with the Alzheimer’s card, and I can display it to the shopkeeper or to one of these major stores I’m in, and they can get in touch with the hospital.” (ID10, Male, PLWD, 72)*
Remaining independent, or supporting some to be	*“I shop regularly, I go out. It’s important to mix with other people, to stay social. Otherwise, one again becomes isolated. And it’s very important to keep one’s social skills and also it keeps me moving, active. And it’s very important. If you don’t practice you lose it. So that’s, that’s the main approach. So I have to keep up with all the activities I’ve done before as much as I can”. (ID19, Male, PLWD, 77)* *“Financially, he couldn’t even begin to sort out at that point. And I actually had to take his card off and so because I have got his power of attorney. So at that point it became totally unreasonable to expect him to deal with that. So shopping was nightmare.” (ID18, Female, Carer, 76)* *“But if you put a trolley in front of her as we did this morning, she can quite happily push around the supermarket, choose the things she wants from the list that she’s made. And maybe I’ll throw in a few items as well. But you wouldn’t notice anything different really.” (ID28, Male Carer, 58)*
Theme 3: Moving to digital: Barriers and facilitators	
Benefits of moving online	*“She used to do her shopping and she used to use a card. And so, I don’t know really, she just used to use our credit or a debit card. So I’m not sure whether she had any problems because she would just go and get a supermarket trolley with food or whatever. And then she would just use a card to pay for it. So she found she could do that. She could remember the pin number as it was then, because obviously it was before contactless.” (ID4, Female, Carer, 64)*
	*“But do you know what? Cashless you know, the contactless card? Yeah, they are good, I have to say… I never use cash.” (ID26, Female, PLWD, 62)* *“I was always doing online shopping before the pandemic, because it’s easier when you don’t drive, especially where I live. This is easier to do online. I’m not having any problems really doing that.” (ID02, Female, PLWD, 52)* *“We’ve always had a joint account. So I actually haven’t had too much problems with that. I haven’t had to use my Power of Attorney or anything because your accounts are joint. So in all honesty, that’s just carried on with direct debits, and pretty much as normal.” (ID21, Female, Carer, 62)* *“So in terms of paying bills, and things like that, again, I’d simplified [the parents’] life because our family history is such that I used to look after my grandmother, she didn’t have Alzheimer’s or dementia or anything, she was just very elderly, you know, well into her 90s. So I’d made everything in her life very easy in terms of putting direct debits in place and everything, you know, being done and being streamlined from that point of view. So I’ve done the same with them [the parents].” (ID24, Female, Carer, 48)* *“I think, with the pandemic, there was [sic] all scare bits, probably true or untrue, about being careful touching hard objects, such as coins, and even the modern £5, £10, £20 notes. They have plastic finish now and the possibility of contracting COVID off that is… I had to get used to virtually having no cash and no notes on me, and totally using a debit or credit card. But I’ve got used to it now and I would, regardless of COVID, I’ll probably carry on doing that now”. (ID10, Male, PLWD, 72)*
Disadvantages of digital finance handling	*“Mummy has never done online shopping. Anyway, she’s in her late 80s. And she never had a computer, she never used one. And so we’ve never had that experience.” (ID01, Female, Carer, 56).* *“In a way, [the mother-in-law] did online shopping with me. I sat with and said, “Oh, which ones do you like?” And she chose the very brightly coloured ones, whether she could see those better, you put them on the screen as large as you could so that she could see them. And so she got to choose her own curtains like that. I tried to involve her as much as I could in decisions. Sometimes it was very difficult, but I did realise the importance for her trying to be independent.” (ID05, Female, Carer, 55)* *“I think it’s simply the practicality of COVID just meant that suddenly we were doing online banking, because that was how you did the banking all of a sudden, and [the parents] were not comfortable with online banking because it was an interface that they that neither parent could get used to or felt confident with. I think they’re just sort of they’d heard all this stuff about people being ripped off or, and so I think they were just anxious around it all.” (ID06, Female, Carer, 49)*
	*“The main issue that arose is [partner] making unwise investments. We eventually managed to persuade him to give up his credit cards, so at least he couldn’t go into debt. […] The bank has been hugely helpful when they could be, and managed to persuade him to give up online banking, and we were able to put controls on the debit card. So he can only make in person purchases and write cheques. And I have access to online banking as with the Lasting Power of Attorney, and I try and keep an eye on what he is spending. But we’ve still found he he’s fallen for things”. (ID17, Female, Carer, 65)*
Theme 4: Fast-tracked digitalisation due to COVID-19	
	*“I’ve had to revert to getting my general shopping in online, which whether I use [a supermarket] or [another supermarket], either one has time slots. You can’t always get the time slot which is suitable for what you want. I’ve been not forced, but I’ve got no option but to buy over £40 at a time, otherwise the delivery charges go up quite dramatically”. (ID03, Male, PLWD, 78)* *“we’d do the shopping for both parents. And then it got a little bit more difficult. And we said, “Can we take the pressure off ourselves here?” So we went online, my sister set it up. We went online with [a supermarket]. But was quite difficult to get the slots. That was supposed to be available for vulnerable people and my dad’s in that top category, we couldn’t, we couldn’t get the bloody slots! And so we just have to, you know, make do.” (ID12, Female, Carer, 58)* *“I live on my own. So obviously, that does present problems. I’ve also had a situation where I picked up or not picked up, I acquired bladder cancer some two years ago at the start of the pandemic. And this has necessitated me staying in, I have actually been in apart from about 19 occasions. I’ve been in around 70 weeks of those two years… I have now to rely on having to do my shopping online. I’m managing all my finances online.” (ID03, PLWD)* *“Since having dementia, I’ve a worry of running out of things. And I had trouble with getting cat food from the supermarket because there wasn’t any… And that’s problematic because then, I mean, I’m quite good at finding the cheapest but what they [the cats] like. And that really made things a lot harder than they needed to be. There wasn’t any because people panic buy, and now I have to think outside the box of where to get these things, and whether I would actually get, whether they’ll be delivered.” (ID02, Female PLWD, 52)* *“And we’ve been shopping online since COVID, we had the food parcels, but a lot of the stuff in the food parcels wasn’t really suitable, I think. We’re very grateful for it, because the staple items were good. And the rest of the time we’ve shopped online, but the problem is you’re, you’re in the hands of the supplier, as to whether you’re going to get it or not”. (ID07, Carer, Female, 69)*
Theme 5: Impact on carers supporting someone with finances	
Additional caring tasks	*“In some areas [managing mother’s finances] just gives me another job to do. And it’s an area that I dislike anyway, I particularly hate numbers, I find them quite difficult to navigate. And I’m fine just doing stuff with an app, that’s really easy. But you know, I mentioned about the [bank], things where so bad at that point time, it all was horrible. So it affected me then, because that was just really, really difficult and distressing, and just intensely challenging.” (ID06, Female, Carer, 49)*
	*“Well, [husband] is not able to do anything with the finances at all… There was a general decline in that before he was diagnosed.” (ID07, Female, Carer, 69)* *“Looking back it’s impossible to know when [the difficulties with finance management tasks] started…* S*he gave up, she became very passive, rather than there was a problem of her losing money, she just let me do it.” (ID08, Male, Carer, 69)*
No support	*“I don’t think there is specific support available. You know, Office of the Public Guardian give you information. I’m a health professional anyway, so I knew a bit more about the Mental Capacity Act than others. And I think because I know more about it, I think I get tied up in knots in making a decision that is in his best interest but which is against his wishes. You know. Yeah. But no, no, I wouldn’t say there’s any sort of specific support for that.” (ID17, Female, Carer, 65)* *“And utility companies and all that, no one will tell you what you’re able to sort of access or what you’re not able to. But then, I suppose, then all the way to, like you say comes down to someone changing stuff like that. Or, I suppose, sort of like the information could be made easier to find… like the websites [being] more user friendly, without the jargon being used”. (ID11, Female, Carer, 58)* *“I feel very underwhelmed by the level of information provided by outsiders, charities… And I’m not sure what these charities do, I feel an absolute lack of support. I think if my dad had cancer, I’d be a whole lot more supported, both by the NHS by the social care system, by my GP and by cancer charities.” (ID24, Female, Carer, 48)*
Worry about acceptance from PLWD/Responsibilities	*“she really doesn’t kind of interest herself in it at all. She just said, again, part of the conversation we had today was her saying, I feel absolutely confident in you, in you having access to my bank account, I don’t mind you having my money and managing my money.” (ID06, Female Carer, 49)*

#### Theme 1: The Potential Dangers of Losing Finance Management Skills Early on

Some carers reported their relative with dementia to have started losing the ability to manage finances prior to their diagnosis. This was only retrospectively recognised by carers, and not used as a reason to go and seek a healthcare professional for advice. Losing the ability to manage finances without a diagnosis in place in particular can be dangerous for the person with dementia and any relatives/spouses/younger children affected by one joint bank account for example. One female spousal carer expressed her concern and only retrospective acknowledgement of her husband’s sudden changes to managing their finances, including changing standing orders.

As a result, carers are linked in well into online finance management, be that spousal or adult child carers, to supervise but also enable the person with dementia to continue engaging in finances as much as they can. This was found to be particularly important for those in the earlier stages of the condition, as many had a keen interest in interest rates, when pensions are incoming, or how their money is spent in general. Having a carer involved helped to calm people with dementia in continuing to manage (some) of their finances and provided reassurance.

“he was very meticulous in keeping his accounts and everything else. And suddenly, well, gradually, it became complete disarray. And you'd have papers everywhere, not understanding what was there. And this is his money. It was one of the early warnings that I didn't actually recognise at the time, I thought I didn't clue it into dementia or anything.” (ID18, female carer, 76)

“And that’s one of his big things that he likes to do that he enjoys doing. […] Not that there’s any great amount of interest, but, you know, he knows that his pension’s going in. […] But the worst thing probably is the fact that most of its online. And he forgets his passwords, and gets locked out a lot. So I seem to be forever either resetting them, [or say] don’t let it do it three times wrong. You know, just wait for me to be home or what have you, those are some of the issues. I mean compared to a lot of people of his age, he still manages remarkably well with online banking. But a lot of it now is jointly managed or controlled by myself, to help him really in keeping calm about it.” (ID22, female carer, 64)

#### Theme 2: Face-to-Face Shopping Skills and Experiences

##### Lack of Support and Recognition From Other Shoppers and Staff

Where carers had gone to supermarkets in-person with their relatives with dementia either before or since the pandemic, they could often feel rushed at the till checkout by other shoppers. This was because people with dementia might need slightly longer to manage the shopping and pay for the groceries. Carers also reported what they described as distressing instances of other shoppers and store staff being rude towards the person living with dementia. While many carers wanted to continue to take the person they care for shopping and maintain the established routine, these negative in-person shopping experiences left some carers avoiding going to the supermarket together.

“It’s [shopping in-person] very difficult with dementia, because it was [husband’s] behaviour that changed. And it's not the actual banks or shops themselves, it's the customers within those areas who are, well, some, frankly, were very rude and aggressive and impatient with him. One pushed a trolley towards him, nearly knocked him over… somebody was excessively rude and then [husband] was in tears in the middle of the store”. (ID18, Female Carer, 76)

Carers suggested that better dementia awareness for staff and customers would make their shopping experience more positive and enable them to support the person living with dementia to continue shopping and maintain their independence.

“But the customers need dementia awareness, as I mentioned. You know, it was very important to me that I was able to take him out to do things as long as I could keep him into a routine.” (ID18, Female, Carer, 76)

The social distancing rules in shops applied during the pandemic exacerbated the difficulties experienced by carers and people living with dementia when shopping in-person. Some carers reported little understanding from shop assistants when they tried to explain why they could not leave the person with dementia alone outside and maintain social distance, as the regulations required.

“I think the biggest issue I had… was when we first started doing the shopping during the pandemic… We got to the till, we were sort of like keeping our two metre distance. So the cashier was serving the woman in front of us and we were stood out two metres back. And then she shouted out, “Are you two together?” And I said, “Yes.” And then she said, “Well, you shouldn't be in here. Can one of you go and stand over there?” So I had to sort of like shout in front of everybody, “My husband has dementia!” And so pretty much that's been the worst. You know, the worst case I've had to deal with.” (ID21, Female, Carer, 62)

##### Feeling Rushed and Anxious

The ability to pay at the self-service checkout is particularly important to people with dementia who may not have a carer, as it allows them to remain independent for longer and still manage their shopping – albeit without the rush from other customers. Many people with dementia expressed their distress at getting rushed at the tills, which left people with dementia feeling uncomfortable at times to go shopping. Using the self-service checkout could provide one avenue of relief.

However, at times using self-service check-out tills resulted in the person living with dementia forgetting to scan the item or to pay for the goods and being accused of shoplifting by the supermarket staff. This can leave people with dementia who are managing their shopping on their own left feeling anxious and uncomfortable to return to the supermarket for fear of the same incidence happening again, and lead to unnecessary avoidance of a basic daily activity and part of independence for people with dementia.

“There are some other reasons to pay by card because it's easier. I don’t have to worry about holding up the queues, and people are impatient, and I notice it more since I’ve been disabled that people just are not kind, especially with the pandemic because it hasn't really helped either… If I have to go shopping in the supermarket, I go to self-service so that I don’t hold up anybody. I can be, I can take my time and there's nobody tutting or being behind me, or something.” (ID2, Female, PLWD, 52)

“It’s the people being impatient… So however much, you know, when you pressured at the checkout, I will not leave the checkout, I’ve got to say. I don't care what anybody thinks of me”. (ID26, Female, PLWD, 62)

Where a person living with dementia was accused of shoplifting, they often sought a ‘proof’ of their dementia diagnosis, such as a letter from the person’s GP or Consultant, or the Alzheimer’s Society’s Helpcard. Carrying such documents when shopping reportedly offered a degree of comfort. People felt that someone else would be able to vouch for them and tell the staff at the store about the impact of dementia on memory.

“So unfortunately, [the experience of being accused of shoplifting] left me a little bit fearful. I know it sounds stupid. It's in the back of my mind that I might do it again. Yes, I've no financial problems, I have a debit card, credit card, no financial problems. Never shoplifted in my life before, it's to do with the illness not me. It just completely went out of my mind to pay… And so it makes me a little bit nervous.” **(**ID10, Male, PLWD, 72)

“The consultant kindly gave me a letter to carry around with me that this [forgetting to pay for the shopping] can possibly happen through the dementia. So if it ever happens again, I've got some proof, together with the Alzheimer’s card, and I can display it to the shopkeeper or to one of these major stores I'm in, and they can get in touch with the hospital.” (ID10, Male, PLWD, 72)

##### Remaining Independent, or Supporting Some to be

Despite some negative experiences, many carers and people living with dementia felt that in-person shopping enhanced their social life, enabled them to mix with people and helped to maintain established routines and independence for longer. While some people with dementia and carers, who felt particularly vulnerable due to other health conditions, reported that they would continue shopping online after the restrictions were lifted, many said they would return to in-person shopping as soon as the rules were relaxed.

However, due to the reduced ability to manage payments and adding items into the trolley that were not required, or multiple versions of an item, supporting someone to go shopping in person can be fraught with difficulties and stress for carers. Whilst carers were keen on supporting their relative to remain independent and stay involved in this daily activity, they recognised the severe difficulties and inability to manage on their own, recognising the need for their own input and support. For some carers, this did not seem to affect or distress them and it was part of their routine to go shopping together.

“I shop regularly, I go out. It’s important to mix with other people, to stay social. Otherwise, one again becomes isolated. And it's very important to keep one’s social skills and also it keeps me moving, active. And it's very important. If you don't practice you lose it. So that's, that's the main approach. So I have to keep up with all the activities I've done before as much as I can”. (ID19, Male, PLWD, 77)

“Financially, he couldn't even begin to sort out at that point. And I actually had to take his card off and so because I have got his power of attorney. So at that point it became totally unreasonable to expect him to deal with that. So shopping was nightmare.” (ID18, Female, Carer, 76)

“But if you put a trolley in front of her as we did this morning, she can quite happily push around the supermarket, choose the things she wants from the list that she’s made. And maybe I’ll throw in a few items as well. But you wouldn’t notice anything different really.” (ID28, Male Carer, 58)

#### Theme 3: Moving to Digital: Barriers and Facilitators

##### Benefits of Moving Online

Using a debit card when paying for items can be advantageous as the need for remembering and understanding monetary values can be overcome easily. Since the payment limit of using the tab functioning of debit cards has been raised further to £100, this has been more simplified for people with dementia. However, even before contactless, using a card was described as a key facilitator by both people with dementia and some carers, offering opportunities to remain independent.

“She used to do her shopping and she used to use a card. And so, I don't know really, she just used to use our credit or a debit card. So I'm not sure whether she had any problems because she would just go and get a supermarket trolley with food or whatever. And then she would just use a card to pay for it. So she found she could do that. She could remember the pin number as it was then, because obviously it was before contactless.” (ID4, Female, Carer, 64)

“But do you know what? Cashless you know, the contactless card? Yeah, they are good, I have to say… I never use cash.” (ID26, Female, PLWD, 62)

One person with young-onset dementia spoke about the advantage of online banking and using a debit card, as younger people with the condition have been used to this type of finance management and spending since before their diagnosis. They were more versed in using online banking and shopping online, and were limited users of face-to-face finance management or shopping activities in the first place. This showed the age-related approach to managing finances in dementia, with young-onset people with dementia more adapt and skilled at using online banking and card payments and thus enabling greater independence.

“I was always doing online shopping before the pandemic, because it's easier when you don't drive, especially where I live. This is easier to do online. I'm not having any problems really doing that.” (ID02, Female, PLWD, 52)

“We've always had a joint account. So I actually haven't had too much problems with that. I haven't had to use my Power of Attorney or anything because your accounts are joint. So in all honesty, that's just carried on with direct debits, and pretty much as normal.” (ID21, Female, Carer, 62)

Direct debits are also helpful for carers, as it removes an additional layer of financial administrative duty. They were seen by carers as an effective way to support their older relatives with dementia to manage their finances and pay bills, and avoided having to check whether bills had been paid. In particular, this applied to holders of joint accounts. Carers reported that they did not need to use the Power of Attorney to amend existing standing orders or direct debits and continued with ‘things as normal’, even after the person they cared for could no longer undertake any finance management tasks.

Some people living with dementia who were particularly vulnerable due to multiple health conditions felt that it was safer for them to use a contactless card than to handle cash for fear of contracting COVID-19. They stopped using note and coins during the pandemic and were planning to continue to do so in future, which also indicates another impact of the pandemic of fast-tracking the move away from traditional money usage.

“So in terms of paying bills, and things like that, again, I'd simplified [the parents’] life because our family history is such that I used to look after my grandmother, she didn't have Alzheimer's or dementia or anything, she was just very elderly, you know, well into her 90s. So I'd made everything in her life very easy in terms of putting direct debits in place and everything, you know, being done and being streamlined from that point of view. So I've done the same with them [the parents].” (ID24, Female, Carer, 48)

“I think, with the pandemic, there was [sic] all scare bits, probably true or untrue, about being careful touching hard objects, such as coins, and even the modern £5, £10, £20 notes. They have plastic finish now and the possibility of contracting COVID off that is… I had to get used to virtually having no cash and no notes on me, and totally using a debit or credit card. But I've got used to it now and I would, regardless of COVID, I'll probably carry on doing that now”. (ID10, Male, PLWD, 72)

##### Disadvantages of Digital Finance Handling

Many carers highlighted the disadvantages of online banking and shopping for many older adults living with dementia themselves, as many older adults with the condition were not skilled in using digital means to manage their money or shop for groceries or other items. This left many older adults with dementia unable to engage with digital forms of banking and shopping, and thus in need of a carer to support them with these tasks. Some older people living with dementia never used online services, such as shopping or banking, and carers decided not to access those services at all.

Other cares were keen to support the person with dementia with accessing shopping online as they felt this would help to continue maintaining the person’s independence. This was particularly the case for parents with dementia, as opposed to spouses with dementia, showing that the digital skillsets from the younger adult child carer generation were influencing the desire to make parents with dementia digitally literate as well, but also the different approaches in terms of adult-child versus spousal carer.

“Mummy has never done online shopping. Anyway, she's in her late 80s. And she never had a computer, she never used one. And so we've never had that experience.” (ID01, Female, Carer, 56)

“In a way, [the mother-in-law] did online shopping with me. I sat with and said, “Oh, which ones do you like?” And she chose the very brightly coloured ones, whether she could see those better, you put them on the screen as large as you could so that she could see them. And so she got to choose her own curtains like that. I tried to involve her as much as I could in decisions. Sometimes it was very difficult, but I did realise the importance for her trying to be independent.” (ID05, Female, Carer, 55)

Some older people living with dementia and their older, spousal, carers felt uncertainty and anxiety about online banking. They had little experience of using digital apps and did not trust online financial services. Thus, in some instances, younger carers supported both the person living with dementia and their older carer with setting up and using online banking services safely, again placing more burden and caring role on younger generations.

Where people with dementia were used to using their debit card as opposed to using hard money (coins and bills), this could also act as a disadvantage however. In some instances, carers had to stop their relative with dementia from using their debit card or bank account in general from making unwise financial investments. Carers had to implement spending blocks on the account and card to safeguard and protect the interests of their relative with dementia.

“I think it's simply the practicality of COVID just meant that suddenly we were doing online banking, because that was how you did the banking all of a sudden, and [the parents] were not comfortable with online banking because it was an interface that they that neither parent could get used to or felt confident with. I think they're just sort of they'd heard all this stuff about people being ripped off or, and so I think they were just anxious around it all.” (ID06, Female, Carer, 49)

“The main issue that arose is [partner] making unwise investments. We eventually managed to persuade him to give up his credit cards, so at least he couldn’t go into debt. […] The bank has been hugely helpful when they could be, and managed to persuade him to give up online banking, and we were able to put controls on the debit card. So he can only make in person purchases and write cheques. And I have access to online banking as with the Lasting Power of Attorney, and I try and keep an eye on what he is spending. But we’ve still found he he’s fallen for things”. (ID17, Female, Carer, 65)

#### Theme 4: Fast-Tracked Digitalisation Due to COVID-19

Whilst digital platforms to conduct banking, manage finances, and spend money were available prior to the pandemic, participants highlighted how COVID-19 face-to-face restrictions fast-tracked a move towards digitalisation. Many people with dementia were vulnerable, although not all people received priority status due to their dementia diagnosis. This vulnerability status meant that people with dementia were to avoid going outside, and instead had to rely on digital platforms to buy groceries or leisure items, handle money, or other everyday activities or items.

For some people living with dementia, engaging in online shopping and banking presented few difficulties as it was something they had done prior to the lockdown, and have successfully continued to do so during the pandemic. In addition, some people with dementia who were also living with a serious health condition felt even more vulnerable to the potential impact of a COVID-19 infection, and felt relieved being able to do this task remotely from the comfort and safety of their own home.

“I’ve had to revert to getting my general shopping in online, which whether I use [a supermarket] or [another supermarket], either one has time slots. You can't always get the time slot which is suitable for what you want. I've been not forced, but I've got no option but to buy over £40 at a time, otherwise the delivery charges go up quite dramatically”. (ID03, Male, PLWD, 78)

“we’d do the shopping for both parents. And then it got a little bit more difficult. And we said, “Can we take the pressure off ourselves here?” So we went online, my sister set it up. We went online with [a supermarket]. But was quite difficult to get the slots. That was supposed to be available for vulnerable people and my dad’s in that top category, we couldn't, we couldn't get the bloody slots! And so we just have to, you know, make do.” (ID12, Female, Carer, 58)

“I live on my own. So obviously, that does present problems. I've also had a situation where I picked up or not picked up, I acquired bladder cancer some two years ago at the start of the pandemic. And this has necessitated me staying in, I have actually been in apart from about 19 occasions. I've been in around 70 weeks of those two years… I have now to rely on having to do my shopping online. I'm managing all my finances online.” (ID03, Male, PLWD, 78)

Moving to online grocery shopping at the start of the lockdown meant that often, despite being classed as vulnerable, people living with dementia and carers were not able to access grocery shopping slots as there were few available. Moreover, many reported not being able to buy the brands they usually bought, access cheaper products and shop for bargains. This resulted in an increase in expenditure on essential groceries and could have implications for their general budgeting.

“Since having dementia, I've a worry of running out of things. And I had trouble with getting cat food from the supermarket because there wasn’t any… And that's problematic because then, I mean, I'm quite good at finding the cheapest but what they [the cats] like. And that really made things a lot harder than they needed to be. There wasn't any because people panic buy, and now I have to think outside the box of where to get these things, and whether I would actually get, whether they'll be delivered.” (ID02, Female PLWD, 52)

“And we've been shopping online since COVID, we had the food parcels, but a lot of the stuff in the food parcels wasn't really suitable, I think. We're very grateful for it, because the staple items were good. And the rest of the time we've shopped online, but the problem is you're, you're in the hands of the supplier, as to whether you're going to get it or not”. (ID07, Carer, Female, 69)

#### Theme 5: Impact on Carers Supporting Someone With Finances

##### Additional Caring Tasks

Managing their relative’s finances was an additional caring task, added onto existing support provided for other activities of daily living such as meal preparation, accompanying someone to support groups, social activities, or day care, or personal care. As finance management was not the only task, providing this additional support was perceived as stressful by many carers, adding to their existing time commitments in caring for their relative.

“In some areas [managing mother’s finances] just gives me another job to do. And it's an area that I dislike anyway, I particularly hate numbers, I find them quite difficult to navigate. And I'm fine just doing stuff with an app, that's really easy. But you know, I mentioned about the [bank], things where so bad at that point time, it all was horrible. So it affected me then, because that was just really, really difficult and distressing, and just intensely challenging.” (ID06, Female, Carer, 49)

For some carers, supporting their relative with finance management tasks was a sudden shift, particularly in light of pandemic face-to-face restrictions and the sudden inability to go shopping or handle finances at the bank. For other carers, the task was gradually added to their caring role, so that carers could acclimatise to the task. Nevertheless, some carers perceived this additional role as burdensome, especially with the added responsibility of ensuring enough money was available for their relative different needs.

“Well, [husband] is not able to do anything with the finances at all… There was a general decline in that before he was diagnosed.” (ID07, Female, Carer, 69)

“Looking back it's impossible to know when [the difficulties with finance management tasks] started… She gave up, she became very passive, rather than there was a problem of her losing money, she just let me do it.” (ID08, Male, Carer, 69)

##### No support

Nearly all carers reported no support in managing their relative’s finances. Whilst carers know how to manage their own finances, there are additional layers of management when managing their relative’s finances, including getting Power of Attorney and liaising with the bank about access to a person’s bank account and a separate debit card, as well as receiving copies of any financial communications.

While some support with caring for the person living with dementia was available to most carers, in particular from Admiral Nurses and some dementia charities, none or very little support with taking on financial management tasks was offered by banks, utility companies or any other statutory organisations.

Carers expressed a wish for readily available finance management support from organisations, to help them handle this additional task. Some pointed out that there is more support for people living with cancer than people with dementia and carers, particularly around managing finances, with people with dementia and carers missing out on vital support for a key daily activity.

“I don't think there is specific support available. You know, Office of the Public Guardian give you information. I’m a health professional anyway, so I knew a bit more about the Mental Capacity Act than others. And I think because I know more about it, I think I get tied up in knots in making a decision that is in his best interest but which is against his wishes. You know. Yeah. But no, no, I wouldn't say there's any sort of specific support for that.” (ID17, Female, Carer, 65)

“And utility companies and all that, no one will tell you what you’re able to sort of access or what you’re not able to. But then, I suppose, then all the way to, like you say comes down to someone changing stuff like that. Or, I suppose, sort of like the information could be made easier to find… like the websites [being] more user friendly, without the jargon being used”. **(**ID11, Female, Carer, 58)

“I feel very underwhelmed by the level of information provided by outsiders, charities… And I'm not sure what these charities do, I feel an absolute lack of support. I think if my dad had cancer, I'd be a whole lot more supported, both by the NHS by the social care system, by my GP and by cancer charities.” (ID24, Female, Carer, 48)

##### Worry AA acceptance From Person With Dementia/Responsibilities

Some carers expressed concern about whether their relative with dementia would be content with the way they were spending their relative’s money for their relative. This was particularly expressed by adult child carers, who experienced a role reversal in suddenly having to care for their mother or father with dementia. This uncertainty and worry left some carers more stressed, by having to take on the additional responsibility of handling the money well. As one carer was deeply worried about this, she had asked her mother with dementia about her perceptions of how her money was handled:

“she really doesn't kind of interest herself in it at all. She just said, again, part of the conversation we had today was her saying, I feel absolutely confident in you, in you having access to my bank account, I don't mind you having my money and managing my money.” (ID06, Female Carer, 49)

## Discussion

This appears to be the first study to show the effects of increased digitalisation on the ability to manage finances and spend money for people living with dementia. Managing finances has been both simplified and further complicated by digitalising how money is spent and managed, with people with dementia and unpaid carers reporting advantages of using direct debits and debit cards, as well as digital illiteracy barriers for older relatives with dementia.

People with dementia are facing an increased digitalisation when managing their finances – from paying with a debit card in shops and using the tab function to having direct debits and managing money online. Research into how people with dementia experience the growing digital interface is limited, with some evidence exploring how people with the condition are engaging with remote support services or time-limited interventions (i.e., Killin et al., 2018; Giebel et al., 2021, navigating; Masoud et al., 2021). Looking specifically at how the pandemic has affected engaging with technologies in dementia, Giebel et al. (2022) showed early on how people with dementia were struggling engaging with digital interfaces to connect with peer support groups or with relatives outside their care home. Seeing peers or family in small Zoom boxes or generally on a screen was difficult to comprehend for many, particularly in the advanced stages of dementia, leaving remote digital connecting with others as a life line but not replacing the benefits of face-to-face interaction and support. Findings by Talbot and Briggs (2022) corroborate this whilst also painting a more positive picture of how people with mild to moderate dementia have engaged with digital technology during the pandemic. It helped reduced social isolation and enhanced well-being, whilst being subject to cognitive fatigue and interface difficulties. Evidence on the wider digital technology usage in dementia therefore suggests advantages, whilst also highlighting multiple engagement difficulties, reflected in this current study.

Highlighting both the advantages and disadvantages of engaging with finances in a digital format, findings from this study suggest that more support needs to be provided. Digital skills need to be improved in the general population, so that if someone develops dementia, they are more skilled at managing their finances digitally without having to learn a new skill. Learning new skills in dementia or other cognitive disorders is difficult and complicated, although not fully impossible in the early stages, likely depending on the dementia subtype (De Wit et al., 2021; Kerkhof et al, 2021). To complement wider digital literacy training in the general population, people with dementia also need to receive adequate support in managing their finances from the point of diagnosis. This should entail support with setting up direct debits, Power of Attorney, as well as how to handle debit cards and paying for things either in the shop or at home on the computer with their card. More research is needed to report on the development and effects of such an intervention.

The need for support with managing finances, and digital finances, is particularly pertinent for those living alone with dementia. Recent evidence from a dementia research cohort in England, the IDEAL study, has reported approximately one-fifth of people with dementia to live alone (Clare et al., 2020). Those living alone were found to have higher cognitive and everyday functioning skills, mostly female, whilst also experiencing higher levels of loneliness and lower levels of life satisfaction than those living with others, and are also found to have higher unmet needs (Miranda-Castillo, Woods, & Orrell, 2010). Whilst people with dementia who live alone may have an unpaid carer, such as an adult child, to support them, people with dementia living alone may be at increased risk of struggling to managing finances adequately without support. As carers in this study highlighted, some people with dementia were more susceptible to overspending on money or to fraud, which can be of greater risk when living alone, especially without any unpaid carer support. Therefore, people with dementia of any living situation urgently need to be provided with guidance on how to navigate the digital finance landscape and how to manage their overall finances at point of diagnosis.

Providing financial and wider digital literacy support will also support the unpaid carers. Carers experienced managing their relative’s finances as an additional task on top of other supportive tasks they were already doing, such as food preparation, medication management, or other everyday activities. Unpaid carers provide an extensive amount of care to people with dementia (ADI, 2022). The higher the needs of the individual with the condition, and the higher the caring duties, the more likely unpaid carers are experiencing high levels of stress and burnout (Sutcliffe et al., 2017). To date, carers are often neglected after a diagnosis albeit their vital role in enabling someone with dementia to stay as independent as possible at home for as long as possible, resulting in high numbers of unmet needs (Tatangelo, McCabe, Macleod, & You, 2018; Zwingmann et al., 2019). By providing both the person with dementia and the carer with support in managing finances, including how to manage finances for another person and liaising with banks about this, this will alleviate some of the stresses of caring experienced by many carers, potentially impacting on their mental well-being. Considering the increased levels of mental ill-health in carers, particularly since the pandemic (Hanna et al., 2022), any opportunity to improve the well-being of this large unpaid workforce needs to be acted upon.

## Limitations

This study benefitted from a meaningful sample size of unpaid carers and people with dementia being interviewed about their experiences, and transcripts being coded by different team members, including two public advisers who were unpaid dementia carers. Interviews were conducted remotely either via phone or Zoom to enable as many people as possible to participate. However, there were some limitations. We interviewed more unpaid carers than people with dementia, as people with dementia were difficult to recruit and carers were more willing and proactive in participating. However, this offered an insight into financial abilities in the more advanced stages of dementia also by speaking to many carers. Future research should complement this study by investigating financial abilities and management experiences in a larger group of people living with dementia, potentially via a national survey to generate a more representative understanding. This study solely explored the experiences of those living with or caring for someone with dementia, suggesting a need for future research to explore the experiences of those providing or dealing with financial services and interactions, such as banks, the post office, supermarkets, and shops.

## Conclusions

Both people living with dementia and their unpaid carers need to be supported in managing finances from the point of diagnosis. People with dementia, and more broadly older adults without a diagnosis, should receive digital literacy training to enable them managing finances digitally if they wish to do so, such as via apps, direct debits, online shopping, and debit cards. This is particularly relevant as it reduces the potential burden placed on carers when they have to take over the management of bills and general finances after a diagnosis. Future research should seek to develop a digital training intervention for older adults and a finance management support intervention for unpaid carers and people living with dementia, which could be implemented nationally and internationally if effective to tackle the here highlighted support needs.

## Appendix. Topic Guide

### Topic Guide for Carers

Thank you very much for agreeing to take part. Let’s start with your general experiences of caring.

1. To start off, could you tell me a little bit about your caring experiences, how long have you been caring for your relative and what have been your experiences?
2. Thinking about all the different types of daily activities that can be affected in dementia, what would you say has been affected the earliest and what has been affected to the greatest extent? How have these activities been affected?
3. Thinking specifically about finance management, what skills have you noticed to deteriorate and be problematic early on? How has finance management been affected more widely?
PROMPT: Value of money? Banking, such as taking money out? Keeping track of finances? Paying bills? Dealing with banks/insurers/utility companies?
4. What have your relative’s experiences been with going shopping either out and about, or shopping online?
PROMPT: Experience of getting to the shops (using public transport/walking)? Dealing with staff in shops? Paying for goods?
5. What have your relative’s experiences been with participating in local paid leisure activities (such as going to eat out, going to museums, the cinema, the theatre, or a gym/art class)?
PROMPT: Experience of getting to these venues, dealing with staff, enjoying the service and paying.
6. Has the pandemic affected your relative’s ability to shop in person/or online, and manage their money, as well as receiving money?
PROMPT: Keep track of finances? Change to online shopping?
7. Are there any activities that your relative does not participate in due to financial or other barriers?
PROMPT: local authority/council financial support?
8. Does your relative have a Power of Attorney in place? If so, was this set up prior to or as a result of the diagnosis? To what extent has the Lasting Power of Attorney been used and what have your experiences been?
PROMPT: Do you feel you received sufficient support to take on this role?
9. Has your relative’s ability or inability to manage finances, go shopping, or participate in paid leisure activities since the diagnosis affected you or the care he/she receives or has received?
PROMPT: Do you feel adequately supported (e.g.by shops, banks, utility companies, leisure companies) to offer the support (if any) that you provide?
10. Lastly, is there anything else in relation to finance management that you feel we have not covered yet?

### Topic Guide for People With Dementia

Thank you very much for agreeing to take part. Let’s start with your general experiences of dementia.

1. To start off, could you tell me a little bit about your experiences of living with dementia?
2. How have you found that the dementia has affected your ability to initiate and perform daily activities, such as washing, managing your medication, preparing food, engaging with social activities?
3. Thinking specifically about finance management, have you noticed any difficulties? Which day-to-day financial decisions were the first to become difficult?
4. What aspects of financial management do you no longer want to undertake and why not?
5. What have been your experiences with going shopping either out and about, or shopping online? Do you go shopping on the (local) high street or prefer shopping online?
PROMPT: Getting to the shops (taking public transport/walking); Experience of dealing with staff in shops; Changes noticed with services moving online
6. Do you spend money on leisure activities, such as activity groups, eating out, visiting the cinema/museums, doing sports, or travel? What have your experiences been participating in these activities?
7. Are there any activities you would like to participate in but can’t due to financial or other barriers?
8. Has the pandemic affected your spending habits in any way, or the way in which you receive money?
PROMPTS: Outside or online shopping? Do you spend less or more?
9. Do you have a Lasting Power of Attorney in place? If so, when and how was this set up and how did you decide on this?
10. In terms of wider finances and dementia care, what are your experiences of paying for care? Do you pay for any services, such as paid home care or social activities in the community, or do you get financial support from the local authority?
11. Lastly, is there anything else in relation to finance management that you feel we have not covered yet?
